# Prevalence and genetic diversity of *Burkholderia pseudomallei* isolates in the environment near a patient’s residence in Northeast Thailand

**DOI:** 10.1371/journal.pntd.0007348

**Published:** 2019-04-19

**Authors:** Rathanin Seng, Natnaree Saiprom, Rungnapa Phunpang, Christine Joy Baltazar, Sarika Boontawee, Thanatchanan Thodthasri, Wirayut Silakun, Narisara Chantratita

**Affiliations:** 1 Department of Microbiology and Immunology, Faculty of Tropical Medicine, Mahidol University, Bangkok, Thailand; 2 Mahidol-Oxford Tropical Medicine Research Unit, Mahidol University, Bangkok, Thailand; 3 Department of Tropical Medicine, Medical Microbiology and Pharmacology, John A. Burns School of Medicine, University of Hawaii at Manoa, Honolulu, Hawaii, United States America; 4 Department of Clinical Microbiology, Buriram hospital, Buriram, Thailand; 5 Department of Medicine, Buriram hospital, Buriram, Thailand; University of Texas Medical Branch, UNITED STATES

## Abstract

**Background:**

*Burkholderia pseudomallei* is the causative agent of melioidosis, a severe infectious disease in tropical regions. It is necessary to understand the risk of acquiring this infection from the environment.

**Methodology /Principal Findings:**

The prevalence, concentration and genetic diversity of *B*. *pseudomallei* isolates collected from two sites in Buriram, Northeast Thailand were investigated. Forty-four environmental samples (18 from soil, 14 from rice rhizosphere, and 12 from water) were collected; of those 44 samples, 19 were collected from near a patient’s residence and 25 from suspected exposure sites and compared with 10 clinical isolates of the patient. Quantitative culture was performed, and *B*. *pseudomallei* was identified using the latex agglutination test and matrix-laser absorption ionisation mass spectrometry. Genotyping was performed in 162 colonies from clinical (N = 10) and environmental samples (N = 152) using pulse-field gel electrophoresis (PFGE) followed by multi-locus sequence typing (MLST) of the clinical strain. *B*. *pseudomallei* was detected in 11 of the 44 environmental samples (1 from soil, 4 from rice rhizosphere, and 6 from water). The bacterial count in the positive soil sample was 115 CFU/g. The mean concentrations ± SDs of *B*. *pseudomallei* in the positive water and rhizosphere samples were 5.1 ± 5.5 CFU/ml and 80 ± 49 CFU/g, respectively. Six water samples with positive results were collected from a pond and water sources for drinking and daily use. All colonies isolated from the patient shared the same PFGE type (PT) indicating monoclonal infection of ST99. Although the 152 colonies from environmental isolates exhibited 25 PTs, none were identical to the patient’s isolates. PT5 and PT7 were most common genotype among the environmental samples.

**Conclusions/Significance:**

Diverse genotypes of *B*. *pseudomallei* were prevalent in the environment. However, the patient may have been infected with a low-density genotype. Intervention strategies for preventing *B*. *pseudomallei* infection are required.

## Author summary

*Burkholderia pseudomallei*, an environmental bacterium, causes melioidosis, a serious but neglected infectious disease that is endemic in many tropical regions. Infection routes include inoculation, ingestion and inhalation. Several environmental sources serve as niches for persistence, providing a mechanism for further dissemination of the bacterium across distances, increasing the risk of human infection through repeated exposure or consumption. Understanding the exposure-relevant environmental sources and transmission routes is required to prevent and control the infection. The present study demonstrates several environmental samples, including those collected from soil and the rice rhizosphere as well as from various water sources, such as pond, rain, dug wells, and pump wells, from sampling points near the patient’s residence were the reservoirs of *B*. *pseudomallei* with diverse genotypes. This suggests that the patient was exposed to *B*. *pseudomallei* multiple times. Thus, intervention strategies targeted at controlling environmental sources, preventing disease spread, and increasing education measures to reduce *B*. *pseudomallei* infection-related morbidity and mortality in Northeast Thailand are urgently required.

## Introduction

Melioidosis is a fatal infectious disease caused by the Gram-negative bacterium *Burkholderia pseudomallei*. The disease is endemic to Southeast Asia and Northern Australia; however, it is also increasingly recognised in other tropical areas throughout Africa, America and South Asia [1]. Approximately 2,000 culture-confirmed cases of melioidosis per year with a mortality rate of 40% have been reported in Northeast Thailand [2]. *B*. *pseudomallei* infection routes include inoculation during unprotected occupational exposure to soil or water, ingestion of contaminated food or water, and inhalation [3]. Increased risk for melioidosis is associated with the following factors in patients: diabetes mellitus, renal diseases, chronic lung disease, thalassaemia, alcohol consumption, male sex and occupational exposure [4, 5]. Although melioidosis clinically manifests as acute to chronic infection, 85% of the cases present as acute infection and usually progress to life-threatening sepsis, often leading to death [5]. *B*. *pseudomallei* has many virulence factors, and its facultative intracellular nature can allow bacteria to escape immune response and persist in several host cell types; giving rise to treatment difficulties and occasionally causing relapse [6]. Currently, there is no vaccine for melioidosis; however, the disease can be treated with intravenous ceftazidime or meropenem followed by oral trimethoprim–sulfamethoxazole for 3–6 months. However, delay in treatment owing to low awareness, late recognition and misdiagnosis can often lead to poor outcomes [5, 7].

The spread of *B*. *pseudomallei* in environment is a major threat to humans and animals, impacting disease burden and resulting in economic losses. Moreover, the incidence of melioidosis is associated with the prevalence of bacteria in the environment [8]. In Thailand, *B*. *pseudomallei* can be isolated from soil and water samples [8, 9, 10, 11]. In Australia, studies reported that *B*. *pseudomallei* can also be isolated from a water treatment plant, the grass rhizosphere, and the aerial portions of a specific grass, indicating the potential spread of the bacteria via grazing animals or farmers when digesting or making direct contact with a contaminated plant [12, 13]. *B*. *pseudomallei* can also persist in water for long periods [14]. Therefore, several environmental reservoirs may serve as a niche for their persistence, providing a mechanism for the further dissemination of the organism across large distances; this might increase the risk of human infection through repeated exposure or the consumption of contaminated sources.

Information regarding the distribution of *B*. *pseudomallei* in the environments surrounding patients’ residences and evidence that patients acquire infection from these potential reservoirs in Thailand is limited. Previously, some studies have investigated the sources of *B*. *pseudomallei* infection. For examples, five studies used molecular typing methods such as PFGE, multi-locus sequence typing (MLST) and whole genome sequencing (WGS) to address the genetic relationship between *B*. *pseudomallei* clones recovered from patients in Australia and Papua New Guinea and the isolates obtained from environments. Soil, water and air samples collected from suspected exposure sites were identified as the sources of infection [15, 16, 17, 18, 19]. One study on three rice farmer patients in Hainan, China, used MLST and 4-locus multi-locus variable number tandem repeat analysis (MLVA-4); however, they could not identify the environmental source of the infection [20]. In Thailand, although *B*. *pseudomallei* was isolated from water samples collected from a public tap and well water near the patient’s house [10], subsequent WGS analysis could not establish a relationship between the clinical and environmental clones [21].

The prevention and control of *B*. *pseudomallei* infection requires an understanding of the environmental reservoirs and transmission route. The Buriram province, where our research team found 114 cases in 2018, is a new hot spot for melioidosis in Northeast Thailand. Here, we hypothesised that the environment surrounding the patient’s residence and the suspected exposure sites are reservoirs and sources of *B*. *pseudomallei* infection. To investigate this, we isolated *B*. *pseudomallei* from a patient and from multiple suspected environmental sources in the Buriram province. The environmental sources included soil, water, and the rice rhizosphere. PFGE and MLST was used to assess the genetic diversity and to identify the infection source. Insights gained from this investigation will be useful for future active surveillance projects aimed towards devising a strategy for the prevention of melioidosis.

## Methods

### Ethical approval

The Ethics Committee of the Faculty of Tropical Medicine, Mahidol University (approval number, TMEC 18–032) and of the Buriram Hospital (approval number, BR 0032, 102.3/57) approved this study and the experimental procedures used herein. Written consent was obtained from the included patient prior to study initiation.

### Patient and identification of *B*. *pseudomallei* from clinical samples

Inclusion criteria was an adult male or female patient (age ≥ 18 years) admitted to Buriram hospital in 2018 with any specimens taken from any sites positive for *B*. *pseudomallei* and be able to provide the informed consent. Isolation and identification of *B*. *pseudomallei* from clinical specimens were performed at the Buriram Hospital using Gram staining, immunofluorescence assay (IFA), latex agglutination and standard biochemical tests [22, 23, 24]. Ten colonies were isolated and collected from the primary cultures of blood (N = 5) and pus (N = 5). The Faculty of Tropical Medicine, Mahidol University, Bangkok further confirmed the bacterial identification using matrix-laser absorption ionisation mass spectrometry (MALDI-TOF MS), as previously described [25]. The bacteria were sub-cultured on Ashdown selective agar plates and incubated at 37°C overnight and then maintained at −80°C in typticase soy broth containing 20% glycerol.

### Environmental sampling

In July 2018, during the rainy season, 44 environmental samples (18 from soil, 14 from the rice rhizosphere and 12 from water) were collected from two sites: (i) the environment near the patient’s residence (N = 19) and (ii) the suspected exposure sites (N = 25). The suspected exposure sites included a community-dug well and a pond with an adjoining rice paddy located at a distance of approximately 1 km and 0.3 km, respectively, away from the patient’s residence. To obtain more environmental *B*. *pseudomallei* isolates, samples were collected at two time points. Of the 44 samples, 15 from the soil, 6 from the rice rhizosphere and 8 from water were collected together 26 days after admission, whereas the rest of the samples, i.e. 3 from the soil, 8 from the rice rhizosphere and 4 from the water samples, were collected 23 days later. For obtaining greater amounts of *B*. *pseudomallei* for genotyping analysis, all the 4 water samples collected the second time were obtained from previously positive water sources.

Of the 18 soil samples, 13 were collected from within a 300-metre radius of the patient’s house and 5 were collected from the pond edge or rice paddy field to which the patient was suspected to be exposed. All the rice rhizosphere samples were collected from the patient’s rice paddy field. During sample collection, all soil and rice rhizosphere samples were not submerged into water. The 12 water samples collected were from the pond (N = 3), rice paddy field (N = 2), community-dug well (N = 1), rain barrel (N = 2), pump well jar (N = 2), and dug well water bucket (N = 2).

Soil sampling was performed as described previously [9]. In brief, a 5–30-cm deep hole was dug using a clean spade and 100 grams of soil was then placed into a clean plastic bag. The bag was sealed and stored away from direct sunlight at an ambient temperature until transported to the laboratory within 2 days. The utensils to be used for soil sampling were washed by rinsing them with bottled water to remove visible debris, cleaned with 70% ethanol and air-dried between each sample collection.

For water sampling, sterile bottles were submerged approximately 10 cm below the water surface to collect 50 ml of water. The collected samples were then stored at an ambient temperature and subsequently transported to the laboratory within 2 days.

For the rice rhizosphere sampling, randomly selected plants were dug out and gently shaken to get rid of the excess soil. Closely adhering soil was considered a part of the rice rhizosphere, as previously described [26, 27]. The rice rhizosphere was carefully excised from the plant and stored in a sterile plastic container. The samples were stored at an ambient temperature and transported to the laboratory within 2 days. The sampling points were tracked using GPSMAP 60CSx. Maps of the study region were created using ArcGIS software version 10.3.1 and LandsatLook Viewer (http://landsatlook.usgs.gov/).

### Culture and identification of *B*. *pseudomallei* from the environment

Soil culture was performed as previously described [9, 28]. Sterile water (100 ml) was added to the 100-gram soil samples, mixed and incubated at room temperature overnight. The water samples were centrifuged at 5,000 rpm (2,800 ×g) for 20 minutes and 500 μl of the reconstituted pellet was collected. The rice rhizosphere samples were cut into small pieces (0.2–0.7 cm) and mixed in two volumes of sterile water by vortexing for 3 min. Two aliquots of 10 and 100 μl each of the soil supernatant, pelleted water and rice rhizosphere suspensions were spread onto Ashdown selective agar plates and incubated at 42°C for 48 h. The resultant *B*. *pseudomallei* colonies were then counted.

All the environmental primary colonies suspected as *B*. *pseudomallei* were chosen from each positive plate for identification using the latex agglutination test [23]; the results were confirmed using MALDI-TOF MS [25]. Ten representative colonies of environmental *B*. *pseudomallei* from each positive plate or all the colonies available on the plates containing <10 colonies were collected for further genotyping analysis from the first collection time. All the environmental colonies from the positive plates collected at the second visit were also genotyped. The bacteria were sub-cultured on Ashdown selective agar plates and incubated at 37°C overnight and then maintained at −80°C in typticase soy broth containing 20% glycerol.

### Bacterial genotyping

Using *SpeI* as a restriction enzyme, PFGE was performed on 10 isolates from the patient and 152 environmental isolates obtained from representative colonies [9]. The genetic relatedness between colonies was analysed using the BioNumerics software version 7.6 (Applied Maths, Belgium). Isolates with identical PFGE patterns were defined as having the same genotype, whereas isolates displaying one or more bands with different molecular lengths were regarded as having different PFGE types (PTs).

Whole genome sequencing (WGS) was performed to obtain the ST of a single clinical *B*. *pseudomallei* isolated from blood. Bacterial genomic DNA was extracted using QIAamp DNA Mini Kit (Qiagen, Germany). The 150-base-read library was prepared with Ion Xpress Plus Fragment Library Kit (Life Technologies, CA, USA) and the sequencing was performed on an Ion Proton system (Life Technologies, CA, USA). The short reads were mapped to the reference *B*. *pseudomallei* K96243 genome using CLC genomic workbench version 12.0 (CLC Bio-Qiagen, Aarhus, Denmark). Sequences of each MLST loci were achieved from the resequencing analysis. Allele and ST were identified using the MLST database (http://bpseudomallei.mlst.net/).

### Statistical analysis

Data were analysed using the GraphPad Prism 6 software (GraphPad Inc.). Unpaired t-test was performed to analyse the differences of the mean of bacterial counts between *B*. *pseudomallei* positive water and rice rhizosphere samples. Fisher’s exact test was used to analyse the prevalence of *B*. *pseudomallei* among different sample types.

## Results

### Patient characteristics

**Fig 1:**
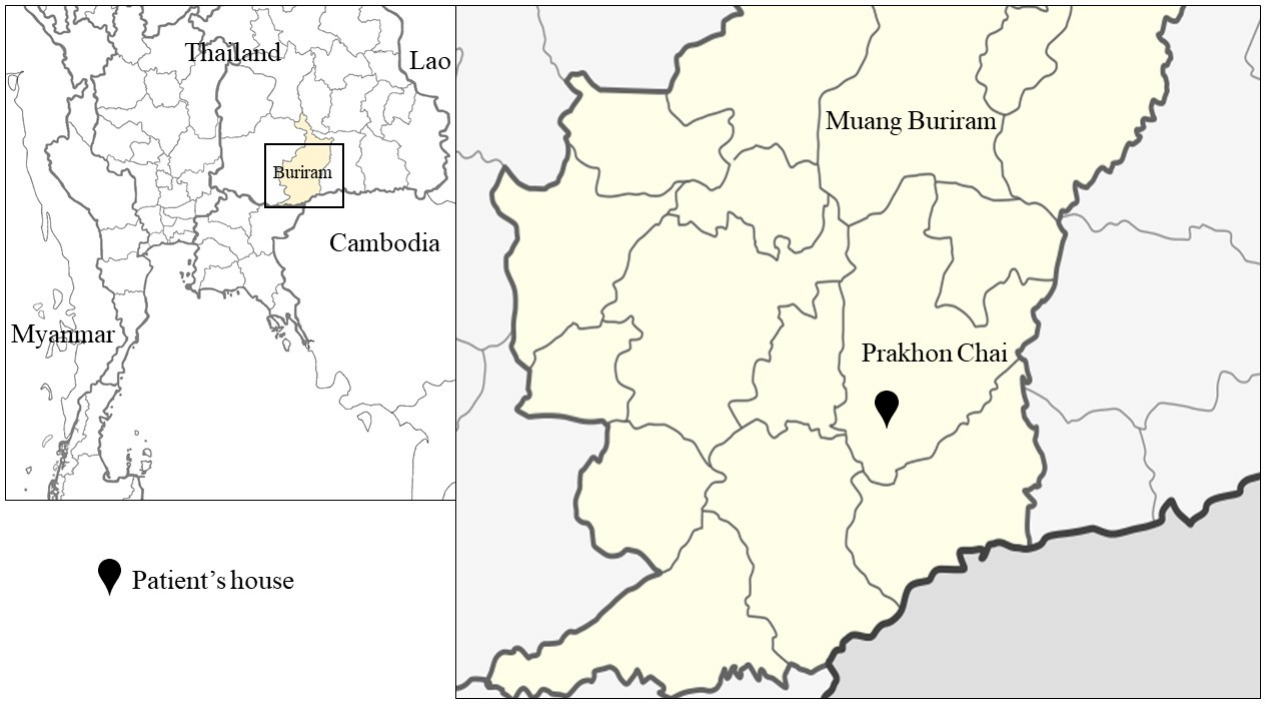
Location of the patient’s residence in Buriram, Northeast Thailand. Maps of the study region were created using ArcGIS software version 10.3.1.

**Fig 2:**
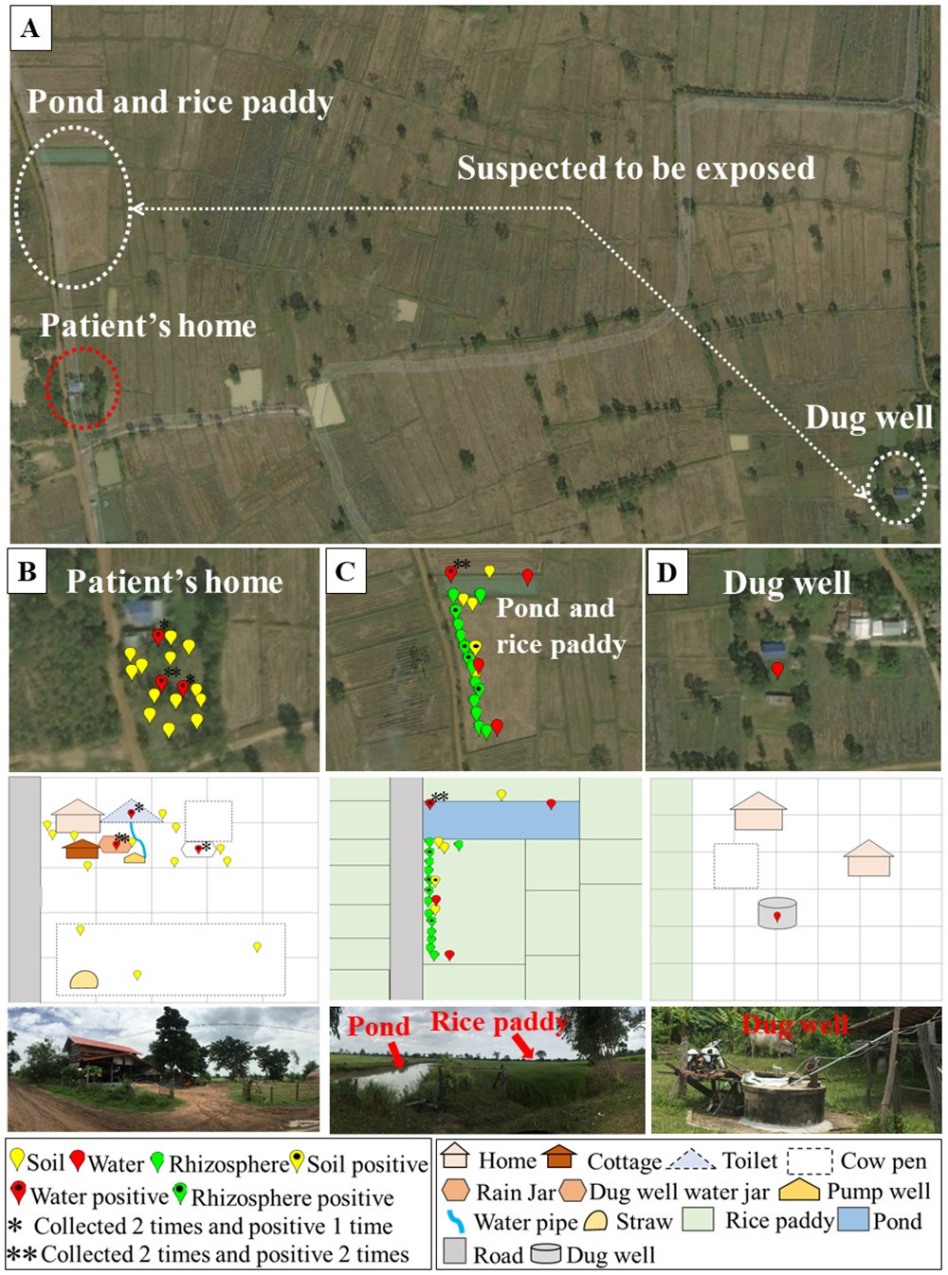
Geographic location of the sampling sites and points. (A) The locations at which the patient was suspected to be exposed (suspected to be exposed), the patient’s house (patient’s house) and the community-dug well used by the patient and community for drinking and cooking (dug well). (B) The pins denote the sampling points for the locations near the patient’s house. (C) The pins denote the sampling points around the pond and the rice paddy field to which the patient was suspected to be exposed. (D) The pins denote the water samples and the enclosed area surrounding the dug well. The sampling points were tracked using GPSMAP 60CSx, and the map was created using LandsatLook Viewer (http://landsatlook.usgs.gov/). Pins with black spots represent *B*. *pseudomallei* culture-positive samples.

A 53-year-old Thai male patient with melioidosis and diabetes mellitus who was admitted to the Buriram Hospital, Northeast Thailand, in June 2018 was included in our study. The patient was chosen to the study because he was the first culture-confirmed melioidosis case who met inclusion criteria and provided a consent form to participate in the study. During the month prior to admission, the patient recalled working at his rice paddy located next to his pond, approximately 0.3 km from his house. He also reported using pump well water in the bathroom near his house and using dug well water for drinking. The dug well water was obtained from a community-dug well located approximately 1 km from his house. The pond, rice paddy and community-dug well were suspected to be potential locations for exposure. Fig 1 presents a map showing the location of the patient’s residence. The locations of the pond, rice paddy and community-dug well are presented in Fig 2.

The patient reported severe headache, fever and right hemiplegia for 1 week prior to admission. The patient was initially treated for his right hemiplegia, acute febrile illness, type 2 diabetes mellitus and ischaemic stroke for 7 days. Culture of his blood collected on day 4 after admission was positive for *B*. *pseudomallei*. On the same day as that of blood collection, computed tomography brain scan with contrast was performed and generalised oedema with a small hypodense lesion approximately 1.17 cm in size was observed. Hence, the patient was transferred to the surgical intensive care unit for brain surgery and continued treatment on day 8 after admission. The pus from his brain was collected on the day of surgery; culturing of the pus produced positive results for *B*. *pseudomallei*. The patient was finally diagnosed with brain abscess caused by *B*. *pseudomallei*. Ten colonies of *B*. *pseudomallei* were isolated from his primary blood (N = 5) and pus (N = 5) cultures. He subsequently received 54 days of intravenous antibiotics (12 days of meropenem followed by 42 days of ceftazidime) and was treated with oral doxycycline and trimethoprim/sulfamethoxazole for an additional 6 months. Treatment was administered at the Buriram Hospital for 23 days but was later continued at a community hospital. The last follow-up conducted 5 months after admission revealed that the patient was paralysed and administered oral antibiotics at home.

### Culturing of *B*. *pseudomallei* from environmental samples

**Fig 3:**
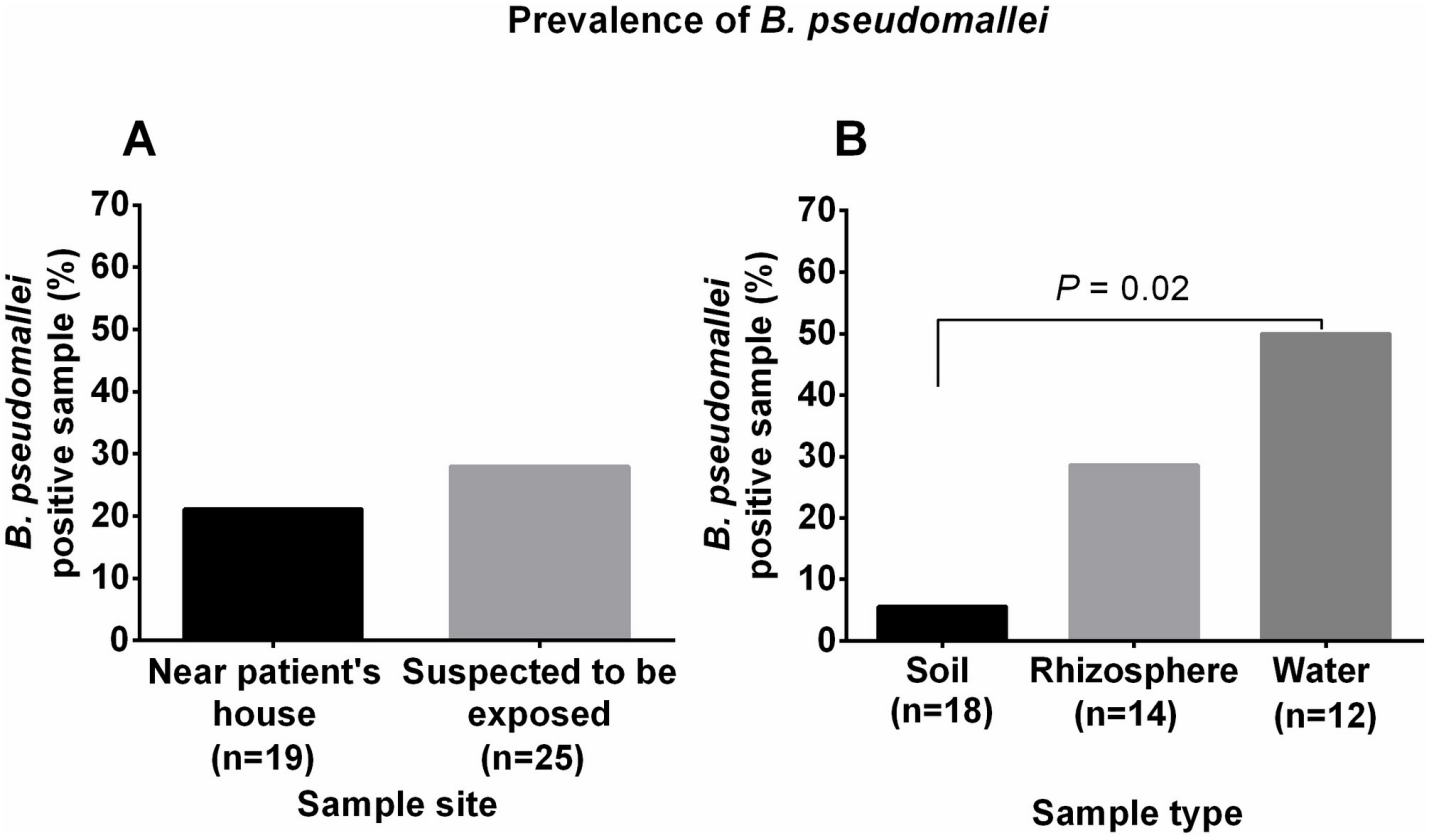
Prevalence of *B*. *pseudomallei* positive samples at two sampling sites among different sample types. (A) Prevalence of *B*. *pseudomallei* positive samples between two sampling sites. (B) Prevalence of *B*. *pseudomallei* positive samples in the soil, rice rhizosphere and water.

To study the distribution of *B*. *pseudomallei* and track the potential sources of infection, 18 soil, 14 rice rhizospheres, and 12 water samples were collected at two different time points from two environmental sites: (i) the environment surrounding the patient’s residence and (ii) the suspected exposure sites (the pond, rice paddy field and community-dug well). The patient lives in the Prakhon Chai district, located 77 km from the Buriram city centre (Fig 1). The distance between the patient’s residence and the pond and rice paddy field was approximately 0.3 km, and the distance between the patient’s residence and the community-dug well was 1 km (Fig 2A). *B*. *pseudomallei* isolates were detected in 11/44 (25%) environmental samples; 7/25 (28%) positive samples were distributed around the suspected exposure sites, and 4/19 (21.1%) positive samples were collected from the environment near the patient’s house (Figs 2B, 2C, 2D and 3A). Overall, *B*. *pseudomallei* was positive in 1/18 (5.6%) soil samples, 6/12 (50%) water samples, and 4/14 (28.6%) rice rhizosphere samples (Figs 2B, 2C, 2D and 3B). The prevalence of *B*. *pseudomallei* in the water samples was significantly higher than that in the soil samples (*P* = 0.02). However, there was no significant difference between the culture-positive proportion of water and the rice rhizosphere (*P* = 0.42) and between the rice rhizosphere and soil samples (*P* = 0.14) (Fig 3B). Of the 6 positive water samples, 2 were collected from the pond and 4 from a rain barrel (N = 1), pump well jar (N = 1) and dug well bucket (N = 2) located near the patient’s house (S1 Table and Fig 2B and 2C). A water sample collected from the community-dug well was negative for *B*. *pseudomallei*.

**Fig 4:**
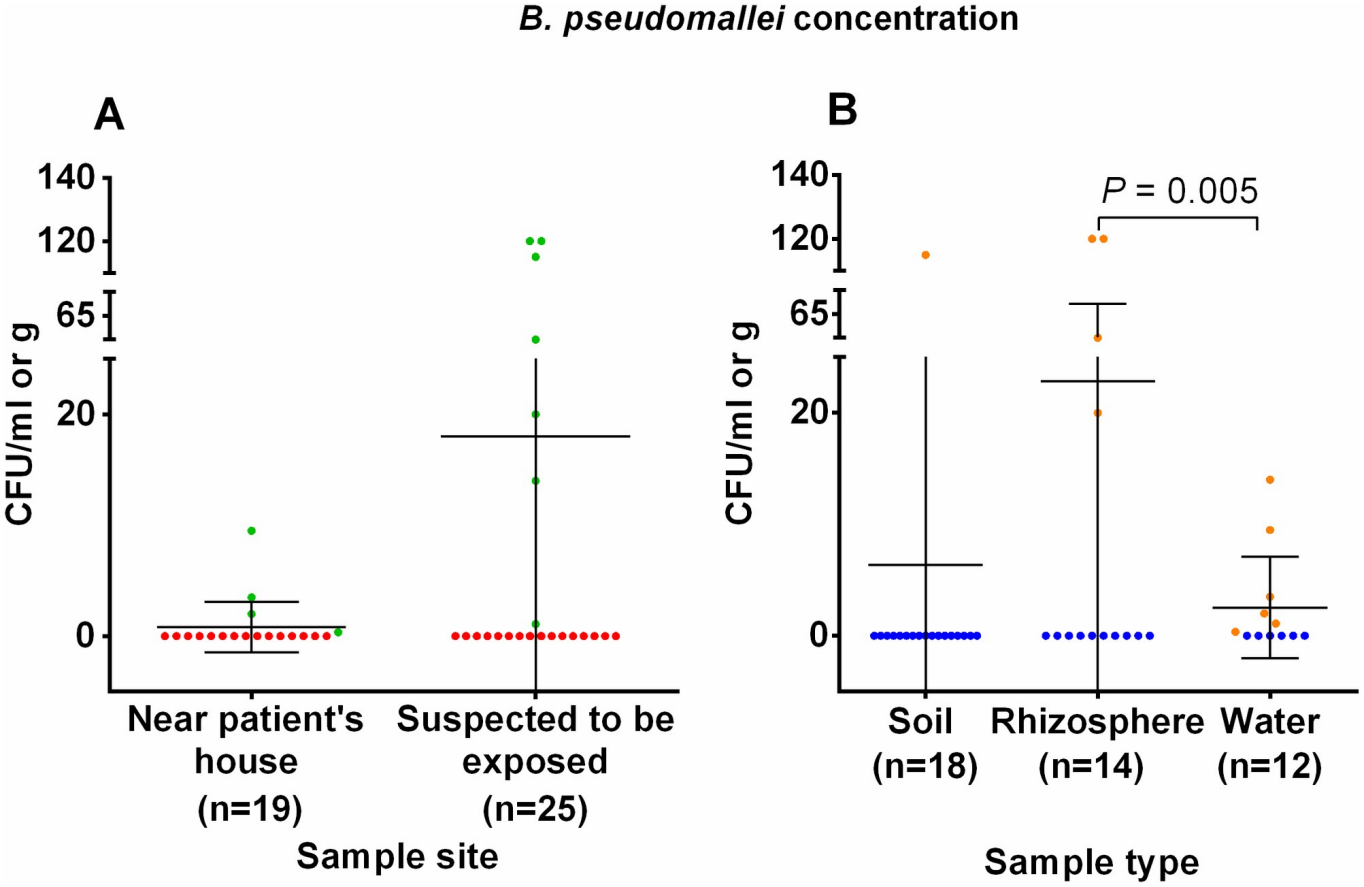
Concentrations of *B*. *pseudomallei* from two sampling sites and three different sample types. (A) Concentration of *B*. *pseudomallei* in all environmental samples from two sampling sites: near the patient’s house and to which the patient was suspected to be exposed. (B) Concentration of *B*. *pseudomallei* in the soil, rhizosphere, and water samples.

The means and standard deviation (SDs) of *B*. *pseudomallei* concentrations from positive samples near the patient’s house (N = 4) and where patient was suspected to be exposed (N = 7) were 11.7 ± 16 CFU/ml and 64.3 ± 54 CFU/g or ml, respectively (Fig 4A). The means and standard deviations (SDs) of *B*. *pseudomallei* concentrations from the positive water (N = 6), rice rhizosphere (N = 4), and soil samples (N = 1) were 5.1 ± 5.5 CFU/ml, 80.0 ± 49 CFU/g and 115 CFU/g, respectively. A high concentration was more commonly associated with the rice rhizosphere samples than with the water samples (*P* = 0.005) (Fig 4B).

### Genotypes of clinical and environmental isolates

**Fig 5:**
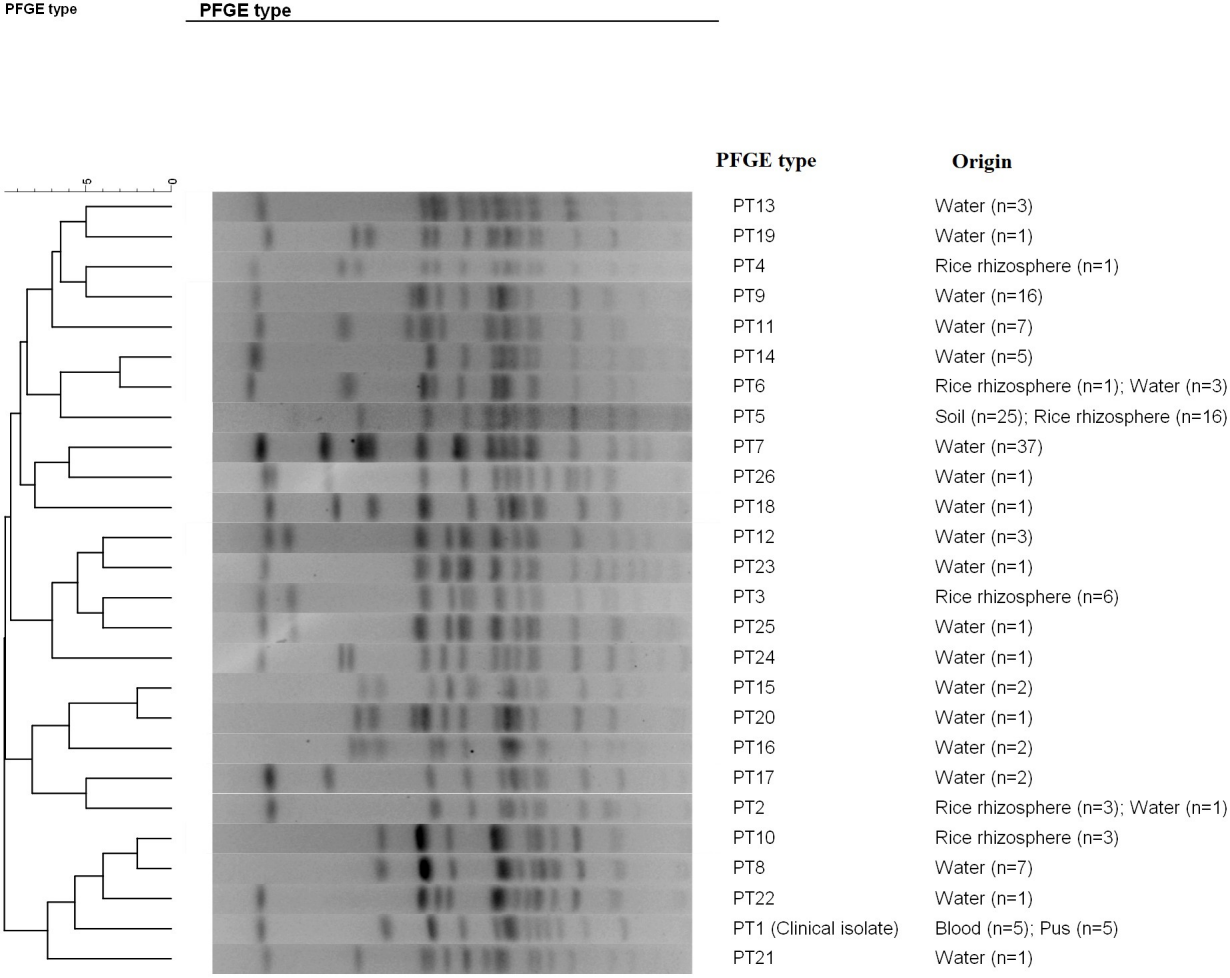
Dendrogram of the 26 representative PFGE types of *B*. *pseudomallei* isolated from the patient (PT1), soil, rice rhizosphere and water (PT2 to PT26) collected from the environment near patient’s residence (PT7, PT8 and PT9) and to which the patient was suspected to be exposed (PT2, PT3, PT4, PT5, PT6, PT7, PT8 and PT10 to PT26) in Buriram, Northeast Thailand. A dendrogram was created using the BioNumerics software (version 7.6).

To identify the environmental source of infection, 10 infected isolates from the patient were genotyped and the results were compared against the 152 environmental isolates obtained from representative colonies collected after culture (Table 1). All the patient isolates shared the same PFGE type (PT1), suggesting infection from a single clone. Further analysis by MLST revealed that this clinical isolate belonged to ST99. Analysis of the 152 environmental isolates demonstrated 25 different PTs. However, none of the environmental isolates shared the same PT as that of the clinical clone (Table 1 and Fig 5). Of the 25 environmental PTs, 20 (80%) belonged to the pond water sample 1W02 (Table 1). Only three other PTs were observed in the other samples obtained from the rain barrel, dug well water, pump well water, soil and rice rhizosphere.

**Table 1:** PFGE patterns of *B. pseudomallei* isolated from the patient and environmental samples and number of isolates for each PFGE pattern and each sample.

PFGE pattern	Patient	Soil sample	Rice rhizosphere samples				Water samples				Total (n = 162)
	Blood (n = 5) Pus (n = 5) (n = 10)	S17 Rice paddy (n = 25)	1R03 (n = 10)	1R04 (n = 3)	1R06 (n = 1)	1R09 (n = 16)	1W02 Pond (n = 51)	1W03 Rain water barrel (n = 10)	1W04 Dug well water bucket (n = 26)	1W05 Pump well water jar (n = 10)	
**PT1**	10 (100%)										10 (6.2%)
**PT2**			3 (30%)				1 (1.9%)*				4 (2.5%)
**PT3**			6 (60%)								6 (3.7%)
**PT4**			1 (10%)								1 (0.6%)
**PT5**		25 (100%)*				16 (100%)*					41 (25.3%)
**PT6**					1 (100%)		3 (5.8%)*				4 (2.5%)
**PT7**							10 (19.6%)	10 (100%)	10 (38.5%)	7 (70%)	37 (22.8%)
**PT8**							4 (7.8%)*			3 (30%)	7 (4.3%)
**PT9**									16 (61.5%)*		16 (9.9%)
**PT10**				3 (100%)							3 (1.9%)
**PT11**							7 (13.7)*				7 (4.3%)
**PT12**							3 (5.8%)*				3 (1.9%)
**PT13**							3 (5.8%)*				3 (1.9%)
**PT14**							5 (9.8%)*				5 (3.1%)
**PT15**							2 (3.9%)*				2 (1.2%)
**PT16**							2 (3.9%)*				2 (1.2%)
**PT17**							2 (3.9%)*				2 (1.2%)
**PT18**							1 (1.9%)*				1 (0.6%)
**PT19**							1 (1.9%)*				1 (0.6%)
**PT20**							1 (1.9%)*				1 (0.6%)
**PT21**							1 (1.9%)*				1 (0.6%)
**PT22**							1 (1.9%)*				1 (0.6%)
**PT23**							1 (1.9%)*				1 (0.6%)
**PT24**							1 (1.9%)*				1 (0.6%)
**PT25**							1 (1.9%)*				1 (0.6%)
**PT26**							1 (1.9%)*				1 (0.6%)

* Indicates *B*. *pseudomallei* isolates collected at the second visit.

**Fig 6:**
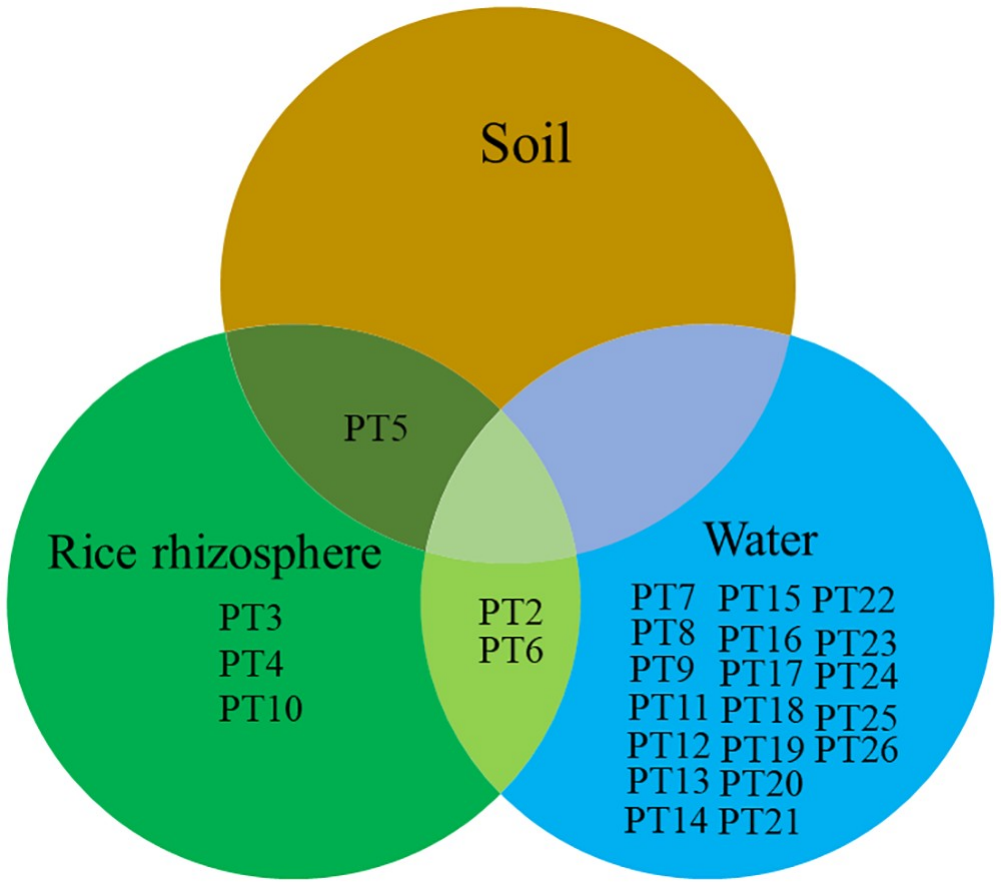
Venn diagram of 25 representative PFGE types from environmental *B*. *pseudomallei* isolates in the soil, rice rhizosphere and water samples. PT7, PT8 and PT9 were collected from the environment near the patient’s residence, whereas PT2, PT3, PT4, PT5, PT6, PT7, PT8 and PT10 to PT26 were collected from the sites to which the patient was suspected to be exposed in Buriram, Northeast Thailand.

Each environmental sample had a predominant type (Table 1). For example, PT3 was found in 60% of 1R03 (rice rhizosphere) colonies, PT5 was found in 100% of S17 (soil sample) and 1R09 (rice rhizosphere) colonies, PT7 was found in 100% of 1W03 (rain water) colonies and 70% of 1W05 (pump well water) colonies and PT9 was found in 61.5% of 1W04 (dug well water) colonies. Only PT2, PT5, PT6, PT7 and PT8 were distributed among more than one sample. PT2 and PT6 were detected in both rice rhizosphere and pond water (Fig 6 and Table 1). PT5 was associated with rice paddy soil and a rice rhizosphere collected from a short distance (40 cm) away, suggesting the same clone was shared in close proximity. Each predominant PFGE clone was found to be individually associated with different sample types (Table 1). However, we observed distinct PFGE groups among different types of environmental samples. PT3, PT4, PT5 and PT10 were distributed among the rice rhizosphere samples, whereas PT7, PT8, PT9 and PT11 to PT26 were distributed among the water samples (Figs 5, 6 and Table 1). Interestingly, PT7 was found in several water samples but was not detected in any of the soil or rice rhizosphere samples (Figs 5, 6 and Table 1).

## Discussion

The high prevalence of melioidosis and an increase in the number of melioidosis-related deaths have been previously reported in Northeast Thailand [2]. Evidence from animal models suggests disease acquisition via inoculation, ingestion and inhalation [29]. *B*. *pseudomallei* present in soil, water, air and food serves as the source of most cases of melioidosis [10, 15, 16, 20].

The present study revealed the high prevalence, quantity and genetic diversity of *B*. *pseudomallei* in the environment surrounding a patient’s residence and other suspected exposure sites, suggesting the possible role of *B*. *pseudomallei* present in the natural environment in the transmission of the infection to humans. The results demonstrate that *B*. *pseudomallei* can persist in many exposure-relevant sources, including soil, the rice rhizosphere, and various water sources such as pond, rain, dug well and pump well water. These water sources have been used by the patient and his family for drinking, cooking, bathing, cow feeding and recreation. Qualitative and quantitative data of *B*. *pseudomallei* in exposure-relevant sources are scarce. Our study also indicates that *B*. *pseudomallei* can be associated with the rice rhizosphere. The mean concentration of *B*. *pseudomallei* in the positive rice rhizosphere samples was significantly higher than that in the positive water samples. While *B*. *pseudomallei* was found to be associated with certain plant species in Australia, such as rice, grass and tomatoes *in vitro* [30], the isolation of this pathogen from the rice rhizosphere in Thailand indicates the high risk of acquiring infection when transplanting the rice seedling or working in the rice field. It is unknown whether the rhizosphere from other local plants in Thailand could also be reservoirs of *B*. *pseudomallei*.

*B*. *pseudomallei* was more frequently detected in water samples than in soil and rice rhizosphere samples. Soil samples were dry around the patient’s house, which may explain why the soil samples tested negative in the present study. Previous studies conducted in the Ubon Ratchathani and Roi Et provinces of Northeast Thailand have also reported the presence of viable *B*. *pseudomallei* in soil and water [9, 11, 31, 32]. The distances between the Ubon Ratchathani and Roi Et provinces and the study sites in this study are approximately 220 and 150 km, respectively. The percentage presence of *B*. *pseudomallei* in uncultivated soil samples collected from Ubon Ratchathani was 80% [9], which was strikingly different from the proportion of positive rice paddy soil samples (28%) collected from the same province [31] as well as the backyard and rice paddy soil samples (26.7%) collected from the Roi Et province, located approximately 170 km from Ubon Ratchathani [32]. Previous studies have also revealed the presence of *B*. *pseudomallei* in paddy rice (60%), bore hole (12%), tap (15%) and well (4%) waters in Ubon Ratchathani, emphasising the need for prevention methods to control the disease [10, 11]. The amount and bacterial concentrations for the positive water samples in this study were lower than those reported for rice paddy water samples in Ubon Ratchathani (prevalence, 50% versus 60%; mean concentration, 5.1 versus 200 CFU/ml, respectively) [11]. In contrast, the amount and bacterial concentrations of positive water samples in the present study were considerably higher than those reported for bore hole water in Ubon Ratchathani (prevalence, 50% versus 12%; mean concentration, 5.1 versus 0.02 CFU/ml, respectively) [10] and accessible water (public tap, well water and spring water) used by households and hotels in Southern Thailand (prevalence, 50% versus 14%; mean concentrations, 5.1 versus 0.03 CFU/ml, respectively) [33].​

PFGE is a reliable method for *B*. *pseudomallei* genotyping within a study [34], although its data could not be used to compare with the clones found by previous studies. Here, PFGE analysis demonstrated the genetic diversity of environmental *B*. *pseudomallei* samples recovered from soil, water and the rice rhizosphere. The predominant genotype of each sampling point was recorded. Our previous study also demonstrated the predominant genotypes in each soil sample collected over a small distance [9]. Predominant genotypes are likely explained by superior biological fitness over other unremarkable types. We postulate that rain and dug well water may provide a mechanism for the dissemination of *B*. *pseudomallei* across the area near the patient’s house and that the same genotypes may become concentrated in the moist soil, rice rhizosphere, or pond. However, in this study, there were no shared PTs between water samples (rain and dug well water) and either the soil or rice rhizosphere. PT crossover was observed within multiple water samples (PT7), between the rice rhizosphere and the soil (PT5), and between the rice rhizosphere and pond water (PT2 and PT6), suggesting that different ecology niches may support the persistence of different genotypes of *B*. *pseudomallei*. To the best of our knowledge, this is the first study demonstrating separate groups of genotypes between the sample types. The ecological factors affecting the absence or presence of those genotypes should be further investigated to better understand the pathogen’s nature in different environmental conditions.

Previous studies reported that the genetic diversity of *B*. *pseudomallei* in soil, bore water, and tank water can range from 1 to 4 genotypes [9, 35]. In this study a similar range of genotypes was found in samples from the rice rhizosphere, rain, pump well, and dug well water, but not in samples from the pond water. A higher diversity of genotypes was observed in the samples from the pond water (20/25 PTs) compared with other water samples. Thus, the diversity of *B*. *pseudomallei* may be supported in a large body of still, natural water but not in smaller water containers. However, to confirm this finding, a larger study is required to investigate the genetic diversity of *B*. *pseudomallei* in a greater number of water samples from different sources and at different time points.

The present study could not establish an environmental *B*. *pseudomallei* clone sharing the same PFGE type as that of the clinical isolates obtained from the patient (PT1). This pathogenic strain may be present in low densities in the environmental samples collected during the study period or the patient might be infected with *B*. *pseuodomallei* in other regions. In addition, it is unknown if diverse genotypes differ in virulence, which might influence successful infection. This clinical isolate was belonged to ST99 which was previously reported in human in Philippines, Thailand, Malaysia and Bangladesh [36, 37]. ST99 was one of the most common stain in Taiwan, found in 24.7% (48/194) of clinical isolates collected during 2004 to 2010 [38]. In Thailand, ST99 was recovered from two soil samples and clinical specimens of two patients in Ubon Ratchathani, Northeast Thailand and in a patient in Songkhla province located in South Thailand [36]. The widespread dissemination distribution of this strain indicated that *B*. *pseudomallei* has frequently dispersed within a short and long distance across Asia continent.

Despite the failure in linking the clinical clone to environmental sources, this study successfully revealed the abundance of *B*. *pseudomallei* in exposure-relevant sites and demonstrated that the soil, rice rhizosphere, and various water types near the patient’s residence in Thailand are major reservoirs for *B*. *pseudomallei*. Therefore, we suggest intervention strategies targeted at these environmental sources; moreover, prevention strategies and increased education measures are urgently needed to reduce the *B*. *pseudomallei* infection-related morbidities and mortalities in Northeast Thailand.

## Supporting information

S1 Table Location and quantitative results of *B*. *pseudomallei* cultures from each environmental sample. (DOCX)
