# Supplementary material for: Prevalence and genetic diversity of Burkholderia pseudomallei isolates in the environment near a patient’s residence in Northeast Thailand
Source: PLoS Negl Trop Dis. 2019 Apr 19;13(4):e0007348. doi: 10.1371/journal.pntd.0007348 (PMC6493765; doi:10.1371/journal.pntd.0007348)
Supplement: S1 Table — (DOCX) [file pntd.0007348.s001.docx]

| Sample No. | Sample type | Location | Source | *B. pseudomallei* culture result |
| --- | --- | --- | --- | --- |
| 1S01 | Soil | Suspected to be exposed | Rice paddy | Negative |
| 1S02 | Soil | Suspected to be exposed | Edge of pond | Negative |
| 1S03 | Soil | Near patient’s house | Near patient’s house | Negative |
| 1S04 | Soil | Near patient’s house | Near patient’s house | Negative |
| 1S05 | Soil | Near patient’s house | Near patient’s house | Negative |
| 1S06 | Soil | Near patient’s house | Near patient’s house | Negative |
| 1S07 | Soil | Near patient’s house | Near patient’s house | Negative |
| 1S08 | Soil | Near patient’s house | Near patient’s house | Negative |
| 1S09 | Soil | Near patient’s house | Near patient’s house | Negative |
| 1S10 | Soil | Near patient’s house | Near patient’s house | Negative |
| 1S11 | Soil | Near patient’s house | Near patient’s house | Negative |
| 1S12 | Soil | Near patient’s house | Near patient’s house | Negative |
| 1S13 | Soil | Near patient’s house | Near patient’s house | Negative |
| 1S14 | Soil | Near patient’s house | Near patient’s house | Negative |
| 1S15 | Soil | Near patient’s house | Near patient’s house | Negative |
| 1S16^*^ | Soil | Suspected to be exposed | Rice paddy | Negative |
| 1S17^*^ | Soil | Suspected to be exposed | Rice paddy | 115 CFU/g |
| 1S18^*^ | Soil | Suspected to be exposed | Rice paddy | Negative |
| 1R01 | Rhizosphere | Suspected to be exposed | Rice | Negative |
| 1R02 | Rhizosphere | Suspected to be exposed | Rice | Negative |
| 1R03 | Rhizosphere | Suspected to be exposed | Rice | 120 CFU/g |
| 1R04 | Rhizosphere | Suspected to be exposed | Rice | 60 CFU/g |
| 1R05 | Rhizosphere | Suspected to be exposed | Rice | Negative |
| 1R06 | Rhizosphere | Suspected to be exposed | Rice | 20 CFU/g |
| 1R07^*^ | Rhizosphere | Suspected to be exposed | Rice | Negative |
| 1R08^*^ | Rhizosphere | Suspected to be exposed | Rice | Negative |
| 1R09^*^ | Rhizosphere | Suspected to be exposed | Rice | 120 CFU/g |
| 1R10^*^ | Rhizosphere | Suspected to be exposed | Rice | Negative |
| 1R11^*^ | Rhizosphere | Suspected to be exposed | Rice | Negative |
| 1R12^*^ | Rhizosphere | Suspected to be exposed | Rice | Negative |
| 1R13^*^ | Rhizosphere | Suspected to be exposed | Rice | Negative |
| 1R14^*^ | Rhizosphere | Suspected to be exposed | Rice | Negative |
| 1W01 | Water | Suspected to be exposed | Pond | Negative |
| 1W02 | Water | Suspected to be exposed | Pond | 14 CFU/ml |
| 1W02^*^ | Water | Suspected to be exposed | Pond | 1.1 CFU/ml |
| 1W03 | Water | Near patient’s house | Rain water jar | 2 CFU/ml |
| 1W03^*^ | Water | Near patient’s house | Rain water jar | Negative |
| 1W04 | Water | Near patient’s house | Dug well water jar | 9.5 CFU/ml |
| 1W04^*^ | Water | Near patient’s house | Dug well water jar | 0.35 CFU/ml |
| 1W05 | Water | Near patient’s house | Pump well water jar | 3.5 CFU/ml |
| 1W05^*^ | Water | Near patient’s house | Pump well water jar | Negative |
| 1W06 | Water | Suspected to be exposed | Rice paddy | Negative |
| 1W07 | Water | Suspected to be exposed | Rice paddy | Negative |
| 1W08 | Water | Community well | Dug well water | Negative |

**^*^** Indicated the samples collected at the second time
